# Development and validation of a novel pulse optimization and beam control system for conventional and ultra high dose‐per‐pulse (FLASH) irradiation

**DOI:** 10.1002/mp.70522

**Published:** 2026-06-12

**Authors:** Luke Connell, Nolan Esplen, Rebecca Lim, Alex Baikalov, Nicholas Coupey, Chinh Nguyen, Emil Schüler

**Affiliations:** ^1^ Division of Radiation Oncology Department of Radiation Physics The University of Texas MD Anderson Cancer Center Houston Texas USA; ^2^ The University of Texas MD Anderson Cancer Center UT Health Houston Graduate School of Biomedical Sciences Houston Texas USA

**Keywords:** beam current transformer, FLASH, UHDR

## Abstract

**Background:**

FLASH radiotherapy requires precise control and minimal variation of dose per pulse (DPP). However, clinical linear accelerators and their beam control systems are designed to ensure accuracy of the temporally integrated dose and do not control for transient variations in DPP during radiation delivery.

**Purpose:**

We introduce a robust external beam control system (EBCS) with radiofrequency optimization and beam monitoring that addresses this need. This system was designed to precisely control the output of FLASH‐capable electron linear accelerators within a clinical range of energies (6–20 MeV) and to monitor the output by using a beam current transformer.

**Methods:**

An EBCS, using either an internal transmission ion chamber or a multistage beam current transformer, was implemented to support delivery of conventional DPPs and ultrahigh DPPs (UH‐DPPs) on a modified clinical linear accelerator. The EBCS was interfaced with the accelerator's gating system, and beam output and stability were maximized by optimizing the accelerating radiofrequency power efficiency through voltage inputs (V_EXT_) to the automatic frequency control interface while the beam was held. The EBCS performance was tested by characterizing the beam‐off latency; beam output stability within and between pulsed deliveries; sensitivity to deviations from optimization solutions; and beam current transformer linearity from conventional DPPs to UH‐DPPs.

**Results:**

The measured beam‐off latency of the system was 56.7 µs (± 4.9 µs). The radiofrequency optimization was shown to reduce the DPP variability within the first five pulses from 26.7% to less than 0.5% for both conventional DPPs and UH‐DPPs. Total output was reduced by up to 20% when V_EXT_ voltage inputs varied from the optimal solution by more than ± 10%.

**Conclusion:**

We developed an EBCS capable of delivering reproducible doses and implemented it on a modified clinical linear accelerator. Through real time readout of the beam current transformer signal and automatic radiofrequency optimization, the uncertainty in DPP within and between each delivery was reduced to < 0.5%, offering unprecedented precision and accuracy.

## 1 INTRODUCTION

Ultra‐high dose rate (UHDR) FLASH radiotherapy (RT) is characterized by the ability to reduce normal tissue toxicity without compromising treatment effectiveness^1^, ^2^, ^3^, ^4^ (the FLASH effect) and is receiving increasing attention. FLASH‐RT is commonly associated with mean dose rates exceeding 40 Gy/s, and the FLASH effect has been reported using both continuous and pulsed sources (electrons, photons, protons, heavy ions).^5^ However, the prerequisite irradiation conditions remain incompletely defined.^2^, ^5^, ^6^, ^7^

Among UHDR‐capable electron sources operating at standard clinical electron energies (6–20 MeV), converted linear accelerators (LINACs) are widely available and use pulsed beams with low pulse repetition frequencies (PRFs) on the order of 10^1^–10^2^ Hz. UHDR delivery is achieved primarily by increasing the instantaneous (intra‐pulse) dose rates (IDR) and dose‐per‐pulse (DPP), such that therapeutic doses may be delivered within a single or small number of microseconds‐long pulses.^1^, ^8^, ^9^ High‐DPP electron sources for FLASH‐RT operating at standard clinical electron energies include commercial systems such as the IntraOp Mobetron (Sunnyvale, CA, USA),^10^, ^11^ the FLASHKNiFE (THERYQ, Rousset, France)^12^ and the Oriatron (PMB‐Alcen, Peynier, France),^13^ as well as modified clinical LINACs converted to exceed conventional output specifications.^14^, ^15^, ^16^, ^17^ Electron UHDRs can also be produced at very high energies (> 50 MeV) using research accelerators such as the Pulsed Energetic Electrons for Research (PEER) system in Australia or the CERN Linear Accelerator for Research (CLEAR) system.^18^, ^19^

These different classes of accelerators differ substantially in beam diagnostics and control architecture, reflecting different primary objectives. Clinical LINAC beam control is designed to deliver a prescribed integrated dose reliably and safely over seconds to minutes, primarily using internal transmission ionization chambers (I‐IC) and associated servos to terminate irradiation when the desired integrated dose is reached.^20^ In contrast, VHEE and high‐energy physics (HEP) research accelerators are designed to preserve beam stability and protect hardware in the presence of high beam power and long transport lines; they therefore rely on broadband diagnostics (e.g. beam current transformers, wall‐current monitors, and beam position monitors) and fast interlocks that can respond on a single‐bunch or single‐pulse basis. Due to the need for very high gradients (> 100 MV/m) and/or long accelerating structures (> 25 m), VHEE systems commonly use sophisticated low‐level RF (LLRF) and feedback frameworks that differ markedly from those of clinical LINACs.^21^ FLASH RT blurs these traditional boundaries between medical LINACs and HEP research accelerators.

Multiple studies have shown that the FLASH effect may depend not only on mean dose rate (MDR) but also on DPP and potentially other irradiation parameters such as total dose, number of pulses, duration of exposure, PRF, pulse width (PW), and IDR, but the extent of these dependencies is unknown.^2^, ^22^, ^23^, ^24^, ^25^, ^26^ Until the relationship between these variables and biological outcomes is clarified, precise control and documentation of all pulse‐resolved parameters are essential for mechanistic studies and for clinical translation.

In standard clinical operation, however, DPP is typically not monitored directly. Clinically approved beam control systems rely on I‐IC signals to monitor integrated dose and dose rate. Thus, large pulse‐to‐pulse fluctuations in DPP may therefore be tolerated if the integrated dose and average dose rate remain within limits.^20^ IEC standards require termination of radiation delivery if the programmed dose exceeds 5% in either the primary or secondary detector, however this is not implemented as pulse‐by‐pulse monitoring. This operational paradigm contrasts with HEP systems, where for example, fast toroidal current transformers and wall‐current monitors routinely provide nanosecond‐scale charge measurements that can trigger beam termination if a single bunch or pulse deviates beyond tolerance^27^, ^28^

For FLASH‐RT, the clinical assumption of temporal averaging no longer holds and when a substantial fraction (or all) of the dose is delivered in one or a few pulses, a single anomalous pulse can produce a clinically meaningful dose error. Accordingly, FLASH‐RT motivates the adoption of HEP‐like, pulse‐resolved charge diagnostics and fast interlock logic, repurposed toward the clinical objective of preventing single‐pulse overdoses while preserving dosimetric traceability.

Consistent with this need, several groups working with converted LINACs have reported a “ramping” effect in which the DPP for the first 2–5 pulses is reduced compared with later “full” pulses.^14^, ^15^, ^16^ This behavior is linked to the automatic frequency control (AFC) system, which tunes RF power and waveguide resonance dynamically while the beam is on. Thus, the initial RF conditions may therefore be suboptimal, leading to for example, the ramping effect. In conventional (CONV) RT, this effect is negligible because thousand pulses are delivered and the relative contribution of early pulses is small. In FLASH, where the DPP is large and the prescribed number of pulses low, uncertainty in the initial pulses can lead to total dose errors that exceed clinical tolerance and may confound biological interpretation if outcome depends on DPP and/or IDR.^26^

An additional challenge arises in high‐DPP beam control when considering the behavior of ionization chambers in UHDR beam, where the simultaneous ionization of many charged particles inside the ion chamber cause the collection efficiency to drop.^29^, ^30^ As a result, new beam diagnostic and control systems are needed if FLASH RT is to be translated into routine clinical practice. For early clinical exploration, the ability to precisely control the number of pulses and the DPP delivered will be essential. However, for broader clinical use, the ability to dynamically control and modify the individual pulses in a train of pulses will be needed. This will require detectors with both high temporal resolution and rapid signal processing.

Beam current transformers (BCTs) have therefore been adopted for UHDR beam diagnostics in electron FLASH systems and may also support beam control.^31^ Prior work has demonstrated a linear relationship between integrated BCT charge and absorbed dose in electron beams,^32^, ^33^, ^34^, ^35^, ^36^ making BCTs a practical, non‐interceptive monitor for pulse‐resolved output. Multistage BCT further enable sensitive operation spanning conventional and ultrahigh DPP on a common platform. However, BCTs lack the capability to provide spatial information about the beam and, like I‐IC, are susceptible to changes in backscatter and irradiation geometry.^19^

In this study we introduce a robust method for UHDR beam optimization and control. An external beam‐control system (EBCS) was developed, and its performance was characterized on a clinical LINAC converted for electron FLASH experiments. The EBCS reliably delivered near‐constant DPP within and between deliveries and ensured accurate delivery of the prescribed number of pulses for both conventional and UHDR modes.

## 2 MATERIALS AND METHODS

### 2.1 LINAC UHDR conversion

A decommissioned Clinac 21EX (Varian Medical Systems, Palo Alto, CA) was converted to deliver both conventional DPP (C‐DPP; < 10^−1^ Gy/pulse) and ultrahigh DPP (UH‐DPP; > 1 Gy/pulse) irradiation as originally described by Schüler et al.^15^ Multiple electron beams of various energies were converted, but only the 16‐MeV electron beam (16e mode) data are described here. The LINAC was operated in service mode, and interlocks related to dose monitoring, beam steering, and dose rate control were disabled. Parameters such as the gun high voltage and grid (clamp) voltage were adjusted on the gun controller hot deck while the beam output was monitored to maximize the DPP and dose rate. The beam was further tuned on the 16e energy card located in the rack‐mounted control chassis beneath the desk, by adjusting gun current (GUN‐I), pulse forming network (PFN), solenoid current (SOL‐I), and the steering (magnet) controls. The carousel was switched to manual mode, and a port cover was removed to allow the user to control the amount of beam scattering (scattering foil selection) or to maximize output without a beam scattering foil. We further implemented a flexible solution that allows the internal transmission ion chamber to be removed from the beam path to reduce beam scatter and further increase DPP and dose rate. The C‐DPP mode of operation was accomplished by reducing the GUN‐I potentiometer setting on the 16e program board.

Active beam monitoring was accomplished by using either the I‐IC or a BCT (ACCT‐S‐055‐H‐CAW1, Bergoz Instrumentation, Saint Genis Pouilly, France) together with an ACCT‐E‐RM‐3R‐3 M‐1000/100/10 mA electronic amplifier (Bergoz Instrumentation, Saint Genis Pouilly, France) connected to a Picoscope 5444D (Pico Technology, Moorpark, CA) computer oscilloscope. The electronic amplifier was configured to have three selectable sensitivity stages (1000 mA/V, 100 mA/V, and 10 mA/V) to allow beam monitoring under both C‐DPP and UH‐DPP. Under UH‐DPP conditions, the I‐IC saturates, such that integrated output is no longer related to dose.^29^ We previously showed that an integrated BCT signal can be used instead to correlate dose for a UH‐DPP beam.^32^ With a rise time of 350 ns and an output resolution of less than 8 µA‐rms depending on the amplification mode, BCTs are a potentially promising beam monitor for UH‐DPP. Although BCTs have been used to record the output of LINACs, we believe this is the first beam control system to directly implement BCTs as beam monitors.

### 2.2 External beam‐control system (EBCS)

An EBCS was developed to enable reproducible delivery of a user‐defined number of pulses on the modified LINAC in both C‐DPP and UH‐DPP modes (section 2.2.1). A pre‐delivery RF optimization strategy was developed to enable consistent and reproducible DPP delivery from the first pulse.

The signal acquisition, RF optimization, and beam control routines were implemented through our EBCS, which was based on a STEMLab 125‐14 (Red Pitaya, Solkan, Slovenia) digitizer board operating at a sampling rate of 125 MS/s. Details of optimization and beam control are outlined in section 2.2.2.

#### 2.2.1 Beam delivery

**FIGURE 1:**
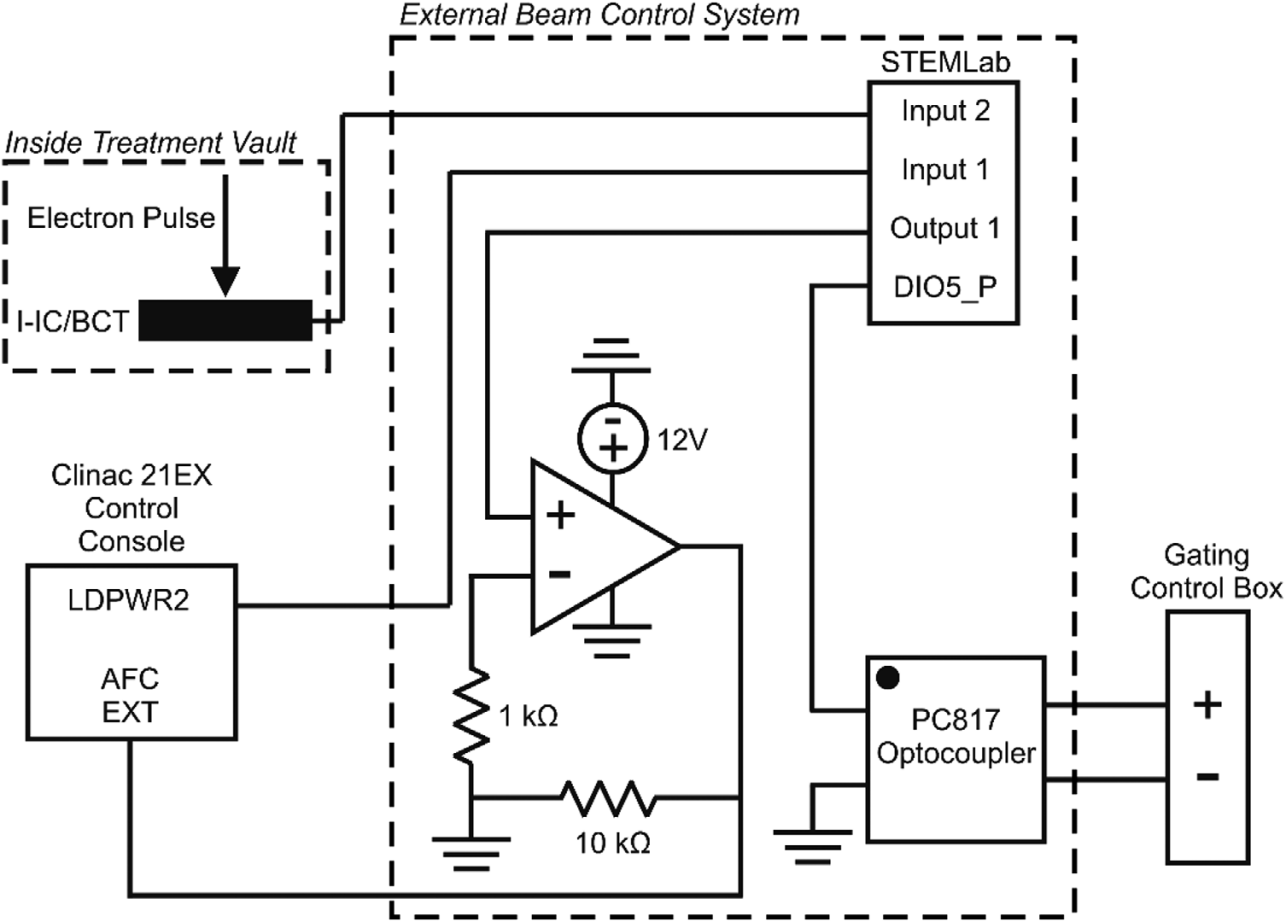
Circuit diagram demonstrating how the external‐beam control system (EBCS) interfaces with the LINAC control console and modified gating control box. The LDPWR2 signal is used by the EBCS to optimize output from the first beam pulse. Electron pulse signals can be received from either the internal ion chamber (I‐IC) or beam current transformer (BCT).

A beam delivery system was implemented by using a modified beam gating box interfaced with a PC817 optocoupler (Bojack, Dongguan Yuhang Electronic Technology, China). Control of the optocoupler, and thus the beam gate, was managed by the EBCS and a rapid pulse counting script that monitored input signals received from either the I‐IC or BCT (Figure 1). When a voltage is applied to the optocoupler, the gating control box is deactivated and the beam turns on, allowing the EBCS to have direct control of the LINAC.

Pulses were detected using a rising edge trigger with a user‐defined trigger threshold setting based on the DPP to be delivered. After a trigger event, the pulse counter is incremented, and the trigger circuit is re‐armed without readout of the data buffer to minimize system latency. After the prescribed number of pulses (N) has been delivered, the EBCS closes the gate and terminates delivery. As an additional level of beam control, a secondary control system was implemented that takes the repetition rate as user input to calculate an appropriate irradiation time (t = N/PRF) and provide a gate timeout that acts as a redundant system for mitigating risk of overdose due to missed pulses. The signal can be split between the EBCS beam control system and the computer used to monitor BCT pulses, therefore a user will still be able to verify that the correct number of pulses was delivered in the event of the secondary system being used to stop the beam. A 5‐V gate signal supplied from one of the STEMLab digital I/O pins was used to toggle between gate opened (beam ON) or closed (beam OFF) states.

#### 2.2.2 Output optimization

For UH‐DPP irradiations that involve fewer than 10 pulses, using the automatic mode of the AFC is prohibitive because of variances in DPP within the first few pulses. The optimizer and pulse delivery system of the EBCS circumvents this limitation by modulating the RF in the accelerating waveguide while the gate is closed (section 2.2.1) and reaching optimal conditions before allowing beam delivery. The radiofrequency modulation is accomplished by supplying an external voltage (V_EXT_) of 0–10 V to the AFC external input. Because the maximum analog output of the STEMLab was 1 V, an operational amplifier circuit was constructed to achieve the desired voltages (Figure 1).

The EBCS uses the reflected RF power (monitored through the Load Power 2 (LDPWR2) signal on Varian Clinac 21EX systems) as input to assess the effect of changes to V_EXT_. The reflected RF power measures the RF energy returning from the waveguide.^37^ Minimizing this reflected power indicates that the waveguide is at resonance and the maximum amount of RF energy is used for acceleration. The circuit diagram for the integrated EBCS and associated I/O is shown in Figure 1.

A gradient descent optimizer was written in Python and run directly on the STEMLab board within the EBCS. The optimizer compares the LDPWR2 signal for a given applied EXT voltage to the optimal LDPWR2 output (baseline) curve, which was determined by manually tuning the LINAC to achieve maximum output and intra‐pulse stability. Gradient descent optimization based on a curve shape similarity metric was used to achieve robust curve matching and minimize loss function. The open‐source Python package *similaritymeasures* module was used for this purpose and the *pcm* (partial curve mapping) function was chosen as providing the best tradeoff between reproducibility and optimizer speed for our system.^38^ After determining the V_EXT_ that minimizes the *pcm* metric, the software moves to a pulse‐counting routine by using the Python application programming interface (API) wrapper.

**FIGURE 2:**
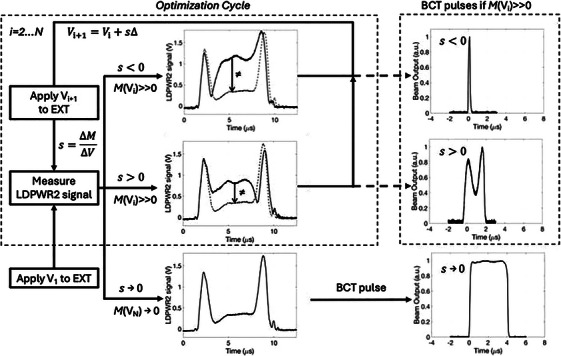
Optimization flow chart for the EBCS indicating the logic and associated signal output. The LDPWR2 signal for a given EXT voltage (V_i_, i = 1…N) is used as input to the optimizer and compared against the ideal curve shape, determined a priori through output tuning to produce maximum output and intra‐pulse stability. When the curve shape metric (M) is minimized ($M(V_{N})\to 0$), the optimization cycle exits, and the beam is delivered (bottom right). Representative electron pulses measured by the beam current transformer (BCT) that correspond to various evaluation points in the optimizer flow are illustrated on the right; only the optimal beam is delivered by preventing beam whenever M is above a predefined threshold. [s = slope from gradient descent optimizer, Δ = variable step size].

To operate the EBCS, the user enters a prescribed number of pulses and then turns on the beam at the console while the gate is closed. The optimizer routine is triggered when the LDPWR2 signal is received at analog input 1, resulting in an initial voltage (V_i_) being sent from analog output 1 to the amplifier circuit before reaching the AFC EXT input. This applied voltage results in a change in the LDPWR2 signal. Thereafter, the applied voltage ($V_{i+1}$) is updated based on the gradient calculated at the previous voltage ($s(V_{i})$), such that $V_{i+1}=V_{i}+s\left(V_{i}\right)\Delta$, where the Δ is the step size (initially Δ = 10^−4^), which dynamically adapts based on the calculated gradient and similarity measure. This process is repeated until the measured signal's similarity measure agrees to within 5% of the acceptance threshold of the algorithm. Once optimization is complete, the script ensures that the same EXT voltage is maintained while the delivery routine (section 2.2.1) is initiated, and EXT is not further modified by the primary pulse‐number based termination system or the secondary timer‐based termination system, which is described below. A visual representation of this workflow is shown in Figure 2, illustrating the LDPWR2 curves and associated electron pulse outputs that would be observed on the computer oscilloscope. In summary, the logic related to impulses and use of the EBCS is shown in Figures 1 and 2. Figure 1 describes the physical circuit diagram of the device, while Figure 2 shows the algorithmic path taken to calculate V_OPT_.

### 2.3 External‐beam control system evaluation

#### 2.3.1 Latency characterization and PRF measurements

The use of time gating as a secondary system to turn off the beam if the primary system fails (section 2.2.1) required that the LINAC PRF be measured for each nominal repetition rate (RR) available in electron mode. To accomplish this, beam was delivered by using the EBCS but radiofrequency optimization was not enabled. The automatic mode of the AFC was used instead and sets of 10 pulses were delivered with a 3‐second interval between sets until the oscilloscope buffer was filled (12–20 sets). The I‐IC signal taken from the TP1 pin on the control console chassis (Figure 1) was recorded with the computer oscilloscope to resolve individual beam pulses. The maximum signal of each pulse was used to determine the time at half‐maximum on the rising edge of each pulse. The difference in time between each successive pair of pulses was calculated, and the inverse of the average was taken to be the measured PRF. For repetition rates that use pulses that are not equidistant, the PRFs within the group (intra‐group) and between groups (inter‐group) were calculated in addition to the mean PRF.

**FIGURE 3:**
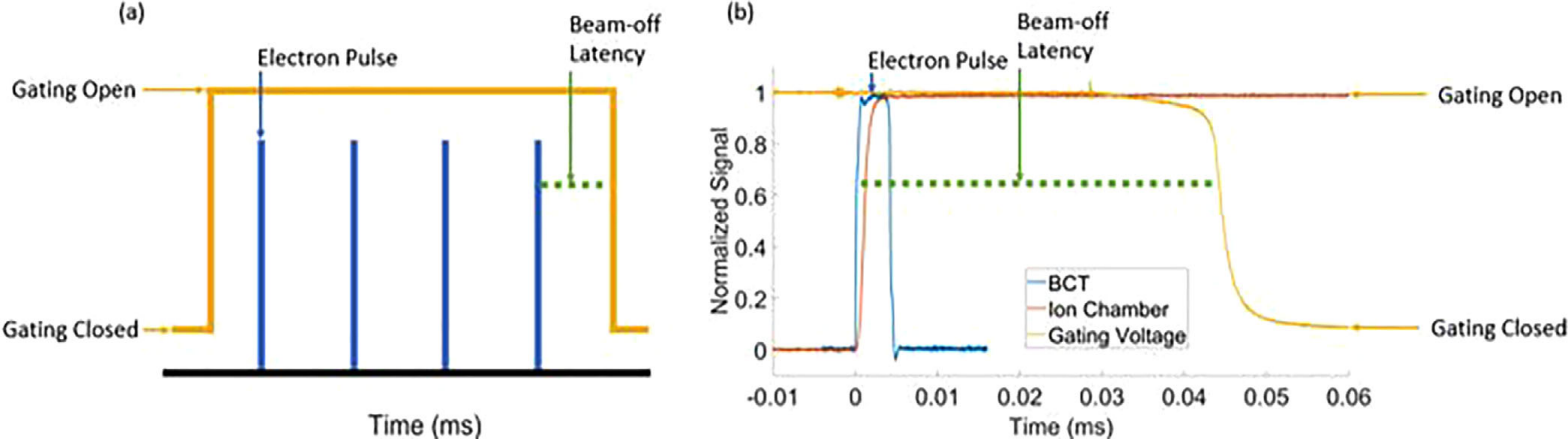
(a) Representative drawing of a pulse train containing multiple pulses, with the gate voltage across the optocoupler shown in yellow. (b) A single electron pulse measured by a beam current transformer (BCT, blue) together with the concurrently recorded internal ionization chamber (I‐IC, orange) Data were acquired in conventional dose‐per‐pulse (C‐DPP) mode to avoid I‐IC saturation and illustrate the relative time scales of the trigger and beam‐off signal latency, and the substantial differences in detector decay time. Although both detector signals decay back to baseline before the next incident pulse, the long ion collection time of the I‐IC makes it appear flat over the timeframe shown. The voltage across the optocoupler is shown in yellow (Gating Voltage); in both cases, electron transport is allowed only while the signal is high (beam ON).

The beam‐off signal latency of the EBCS establishes a practical limit on the system's ability to terminate delivery through the gating interface and was determined by measuring the time difference between the final pulse and the time until the beam off signal is sent. The time of final pulse was defined at the half‐maximum of its rising edge while the time of beam‐off was defined at the half‐maximum of the optocoupler signal falling edge (gate closed). The computer oscilloscope was connected to the output of the BCT and optocoupler gate to view both signals on the same timescale. Five deliveries of 10 pulses each were delivered at each repetition rate. Figure 3 (a) shows a representative diagram of a pulse train, where several electron pulses are bounded by a single open gate envelope, and (b) shows a representative example of the signals measured when the EBCS terminates delivery.

#### 2.3.2 Output stability validation

The DPP stability of the EBCS and effects of the dose servo were assessed first in the pre‐conversion (unmodified) LINAC configuration in the 16 MeV mode. The time‐resolved I‐IC signal was recorded for 5 irradiations of 20 pulses each and subsequently integrated for each pulse within a delivery. Measurements were collected with and without the EBCS optimizer, in each case repeating acquisition with and without the dose servo. The function of the dose servo is to monitor the dose rate within a 50‐ms servo period; if the dose rate within this time scale reaches the target dose rate, then the servo will restrict electron gun pulsing until the servo period restarts.^37^ This procedure was repeated after the UHDR conversion of the LINAC and replacing the I‐IC signal with that of the BCT, which was processed as described by Liu et al.^32^ Because the dose servo can selectively drop pulses, creating unpredictable changes in MDR, the dose servo was inactivated during delivery of UH‐DPPs.

For each delivery, the EBCS attempts to identify the V_EXT_ that minimizes the optimization metric M (i.e., V at min(M)) and thus optimizes beam output and stability (Figure 2). To characterize the performance and requirements on the calculated V_EXT_ at min(M) (V_opt_), the optimization script was modified to send the final voltage as a percentage between 85% and 120% of V_opt_. The V_opt_ itself is a variable quantity that changes between deliveries, emphasizing the importance of having a per‐delivery optimization routine. A selection of 8 voltages between 0.85 V_opt_ and 1.2 V_opt_, 5 trains of 10 pulses each, were delivered to calculate initial (first pulse) and the meanTh output and variance between deliveries.

#### 2.3.3 BCT linearity under optimal radiofrequency conditions

To demonstrate how DPP on the modified LINAC could be changed by using the EBCS, we applied 8 GUN‐I voltages between 14 V and 0.093 V at the 16e energy board and subsequently measured the DPP. Doses were measured by using 2 cm x 2 cm EBT3 Gafchromic films (Ashland, Bridgewater, NJ, USA) at 1.2 cm depth in solid water and a source‐to‐surface distance (SSD) of 98 cm with the 10 × 10 cm^2^ applicator. The film measurement depth was selected to be within the region of dose‐maximum based on previously acquired depth‐dose data at 98 cm SSD for the UHDR beam without scattering foils. At this location and depth, a usable field size (90% isodose line) of 2 cm diameter is achieved. Three films were irradiated for each current setting. Initially, a single pulse was delivered per film, and as the DPP was reduced, the number of pulses was increased to ensure that the total dose was within the usable range of the EBT3 film (i.e., up to 50 Gy^39^). The films were scanned 24 h after irradiation on an Epson (Epson America, Los Alamitos, CA, USA) 12000XL scanner. Three additional blank films (0 Gy) were set aside from the same sheet to allow background correction. Net optical density was measured, and dose was calculated using a calibration curve created from the same batch between 0.5 and 50 Gy, as described previously.^39^, ^40^

The BCT pulse was also recorded to determine the pulse structure as gun current was reduced. For pulses delivered with ≤4 V measured at the GUN‐I potentiometer, the most sensitive stage (10 mA/V) of the 3‐stage BCT was used to ensure adequate signal‐to‐noise ratio at the lowest output. For pulses recorded with GUN‐I ≥8 V, the intermediate stage (100 mA/V) of the BCT was used to avoid saturation of the BCT amplifier. Each pulse was processed and integrated as described in section 2.3.1 to yield the charge per pulse through the BCT. These data were subsequently plotted against the measured GUN‐I voltages to determine the linearity of BCT response across sensitivity modes.

### 2.4 Statistics

The data are shown as the mean value and standard deviation unless otherwise noted. A one‐way analysis of variance between the different repetition rates was used to determine differences in beam‐off latency between the nominal repetition rates used on this accelerator.

## 3 RESULTS

### 3.1 Latency characterization and PRF measurements

The LINAC PRF of each nominal repetition rate setting was calculated using the mean period measured between 10 consecutive pulses (Table 1). Repetition rates 4 and 6 were excluded due to the non‐equidistant pulse delivery for those repetition rate settings. The beam‐off latency, which represents a theoretical limit in PRF that the EBCS can control, was calculated individually for each repetition rate. A one‐way analysis of variance between the different repetition rates revealed a *P* value of 0.245, indicating no statistical difference in beam‐off latency between the different repetition rates. No statistically significant difference in beam‐off latency between the different nominal repetition rate settings was found. The overall mean value across all groups was 56.7 µs ± 4.9 µs.

**TABLE 1:** Mean pulse repetition frequency (PRF) and beam‐off latency at each nominal repetition rate (RR).

RR	Measured PRF, Hz	Beam‐off Latency, µs
1	18.0 ± 0.0	59.0 ± 3.6
2	36.0 ± 0.0	57.1 ± 2.7
3	60.0 ± 0.0	60.2 ± 5.3
5	90.0 ± 0.0	54.2 ± 6.2
7	180.0 ± 0.0	54.0 ± 2.1

Throughout this study, the EBCS successfully delivered the prescribed number of pulses for all nominal repetition rates, with no failures observed for repetition rates 1 through 5; at the highest repetition rate (RR7), however, a rare failure mode (signal leak) emerged caused by an extra pulse being delivered after the beam gate had been closed (gate signal low; Figure 3). We hypothesize that the cause of this failure was the finite gate voltage decay time of the optocoupler. To better characterize this failure mode, an additional 202 deliveries at repetition rate 7 were conducted for a total of 246 deliveries of 10 pulses per delivery. The total failure rate was determined to be 2.4% (6 of 246), amounting to a treatment success rate of 97.6%. At all other repetition rates, no signal leak failures were observed.

### 3.2 Output stability validation

For a train of 20 pulses, the output per pulse was normalized to the average of the final 5 delivered pulses, which has been shown to be a stable, post‐ramping region.^16^, ^41^ For each of the beam control configurations (pre‐conversion without the EBCS, pre‐ and post‐conversion with the EBCS), beam output was determined based on the integrated signal from either the I‐IC (for the pre‐converted LINAC) or BCT (for the pre‐converted and converted LINAC).

Within the first 5 pulses of the delivery, the percent standard deviation for the beam output without EBCS optimization and with the dose servo setting on was 26.70%; that for the beam without EBCS optimization and the dose servo setting off was 4.93%. Using our EBCS, the percent standard deviation in output with and without the dose servo on was reduced to 0.41% and 0.27%, respectively, and percent standard deviation in output after the conversion to UHDR was maintained at 0.40%.

The output per pulse was measured for V_EXT_ values which were set between 85% and 120% of the EBCS optimized value (V_opt_) for a given delivery. The average output of 10 pulses was reported per delivery for 5 deliveries. We observed that for V_EXT_ values that deviated by more than ± 5% from V_OPT_ the beam output was substantially reduced (Figure 5). The reproducibility of the pulse train also suffered at values greater than ± 5% deviation, as demonstrated by the increased output variance.

### 3.3 Output stability and BCT linearity under optimal RF conditions

The DPP was measured for GUN‐I values ranging from an initial value of 14 V to a minimum 0.093 V. The observed DPP values were close to clinical values, approximately 0.1 cGy/pulse, below 4 V, at which point the DPP rapidly increased as the GUN‐I voltage was increased (Figure 6). The maximum value of 16.25 Gy/pulse was observed at a GUN‐I value of 14 V. The decrease in DPP with decreasing gun current, with no further modifications to other beam tuning settings, was shown to be caused by a reduction of the maximum intra‐pulse dose rates within each pulse, characterized by a decrease in pulse height for the same PW. However, pulse shape was also distorted with this approach when lower gun currents were used.

When comparing the pulse‐averaged integrated BCT signal at various gun currents to the DPP, a clear linear relationship could be established. Even if the square pulse shape, and thus intra‐pulse dose rate stability, is compromised, the dose can be recorded and reported accurately.

## 4 DISCUSSION

The EBCS developed in this work performed two key functions: (1) it reduces pulse‐to‐pulse variability within and between deliveries and (2) it enforces accurate delivery of the prescribed number of pulses, with time‐gating redundancy serving as a secondary (back‐up) termination mechanism. These capabilities directly address a central challenge in FLASH‐RT: because mechanistic studies increasingly implicate time‐structure (including DPP) in addition to MDR,^26^ biological interpretation requires that PW, IDR, DPP, PRF, and pulse count be both controlled and documented with pulse‐level fidelity. In the FLASH regime, even a single anomalous pulse can materially alter delivered dose and potentially alter the irradiation conditions relevant to the FLASH effect.

Notably, the EBCS achieves stable waveguide resonance prior to dose delivery by completing RF optimization before beam delivery is enabled. This differs from conventional clinical operation, in which resonance is typically approached during irradiation through the AFC. That approach is adequate for conventional treatments because integrated dose accuracy is maintained through averaging across thousands of pulses. In UHDR delivery, however, variability in the initial pulses can contribute substantially to the delivered dose and can therefore produce clinically meaningful dose errors. By eliminating AFC‐ and dose servo‐derived output variability, the EBCS produced stable DPP for single‐ and multi‐pulse UH‐DPP deliveries and provides a framework for pre‐delivery optimization which ensures consistency in all irradiation parameters relevant to the biology of the FLASH effect.

Time‐varying conditions in the accelerating components between radiation deliveries shifts the V_EXT_ value that minimizes the curve shape metric and corresponds to optimal RF power transfer. Accordingly, the V_EXT_ setpoint that produces resonance is delivery‐specific rather than universal. The EBCS accounts for these variations by identifying, for each delivery, an appropriate V_EXT_ that minimizes the difference between the measured and optimal reflected RF power signals. The sensitivity of beam output to even modest departures from the delivery‐specific optimum (V_opt_) is illustrated in Figure 5; deviations of approximately 5% from V_opt_ produced large (> 20%) output changes and degraded both intra‐train and inter‐train pulse stability. In addition, V_opt_ varied by more than 5% between deliveries, precluding selection of a single fixed setpoint underscoring the need for pre‐delivery optimization. Collectively, these observations indicate that a FLASH‐compatible control approach should (i) determine V_EXT_ on a per‐delivery basis with strict acceptance criteria and (ii) avoid reliance on a universal V_EXT_ setting, representing a marked improvement over strategies that assume time‐invariant RF conditions.

We have previously demonstrated linearity between integrated BCT output (charge) and absorbed dose in an electron UHDR beam,^32^ motivating the use of a BCT with the EBCS as a non‐intercepting beam monitor over a wide range of DPPs. In HEP facilities, BCTs and wall‐current monitors are ubiquitous for bunch charge measurement and fast machine protection; here, the same diagnostic philosophy is repurposed to address the inability of clinically used integrating monitors to provide a sufficient safety margin when dose is delivered in only a few pulses. However, several limitations remain before BCT‐centric monitoring can serve as a clinical‐grade beam monitor. BCTs do not provide spatial dose information, whereas clinical beam monitor systems ultimately require both output and spatial verification. Furthermore, BCT signals can be influenced by backscatter and changes in irradiation geometry and collimation, analogous to internal monitor chamber dependencies.

Characterization of the EBCS included quantifying a known failure mode in which one additional pulse is delivered. Across 246 deliveries spanning multiple pulse counts and repetition rates, the EBCS delivered the prescribed pulse number in all but 6 deliveries at the highest repetition rate (180 Hz), corresponding to a 97.6% success rate. Failures were attributable to optocoupler decay time limitations rather than control logic errors; an extra pulse occurred after gate closure due to the slow fall time of the optocoupler output. Planned replacement with a faster component and circuit refinements to avoid transistor saturation are expected to resolve this limitation and extend accurate operation to higher PRF. If this device is limited to the second greatest repetition rate of 90 Hz, a MDR of 1495.2 Gy/s can still be achieved at the peak DPP.

**FIGURE 4:**
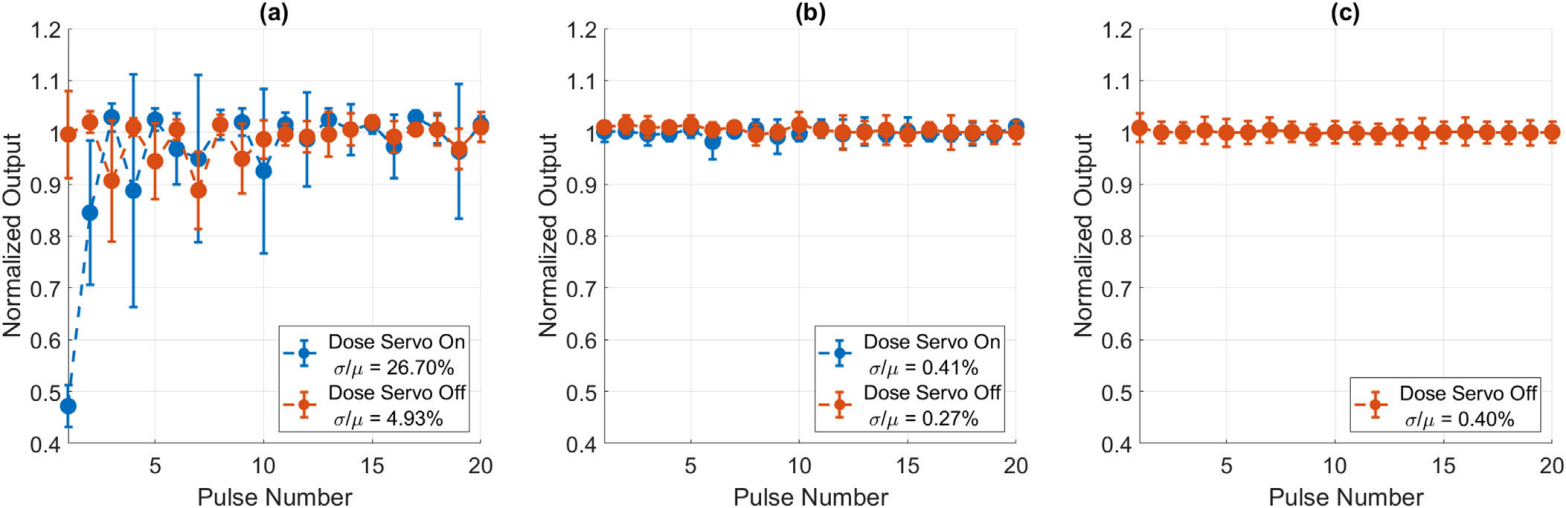
Output per pulse in a 20‐pulse delivery (a) before and (b,c) after LINAC conversion for ultrahigh dose‐per‐pulse delivery, normalized to the average of the last 5 pulses for each data set. Error bars represent the standard deviation of a set of 5 deliveries of 20 pulses each. Output variation with pulse number (a) for the unconverted LINAC with and without dose servo activated and automatic frequency control (AFC) active and (b) using the EBCS beam control routine without AFC before the LINAC was converted to UH‐DPP mode. (c) Output variation with pulse number for the UH‐DPP‐converted LINAC together with the EBCS. σ/μ is the percent standard deviation of the mean (μ) within the first 5 pulses.

It has previously been reported that the gun current setting can be used directly as a means for varying the DPP.^15^ However, an important limitation as we found in our study of using gun current to modulate output and achieve CONV‐like DPP values was the loss of pulse integrity. For a higher gun current, a characteristic square pulse was produced; however, as gun current was decreased, the “center” of the pulse began to “collapse” (Figure 4). The loss of signal, and thus DPP, upon lowering gun current came principally from the temporal center of the pulse, resulting in what functionally appears as two pulses separated by ∼4 µs. Care is thus needed from a beam control perspective when using the gun current setting to lower DPP to avoid double‐counting pulses. This was not a concern for the present implementation because of the finite re‐arming time of the EBCS trigger, which was longer than the width of an electron pulse. However, achieving C‐DPP delivery by simply lowering the gun current might have implications for translational research purposes. Specifically, the effects on pulse shape represent a significant intra‐pulse dose rate variability that is not seen in clinical beams and could have implications for biological effects. Until we know the relationship between specific irradiation parameters and the FLASH sparring effect, this strategy to achieve C‐DPP deliveries should be avoided.

**FIGURE 5:**
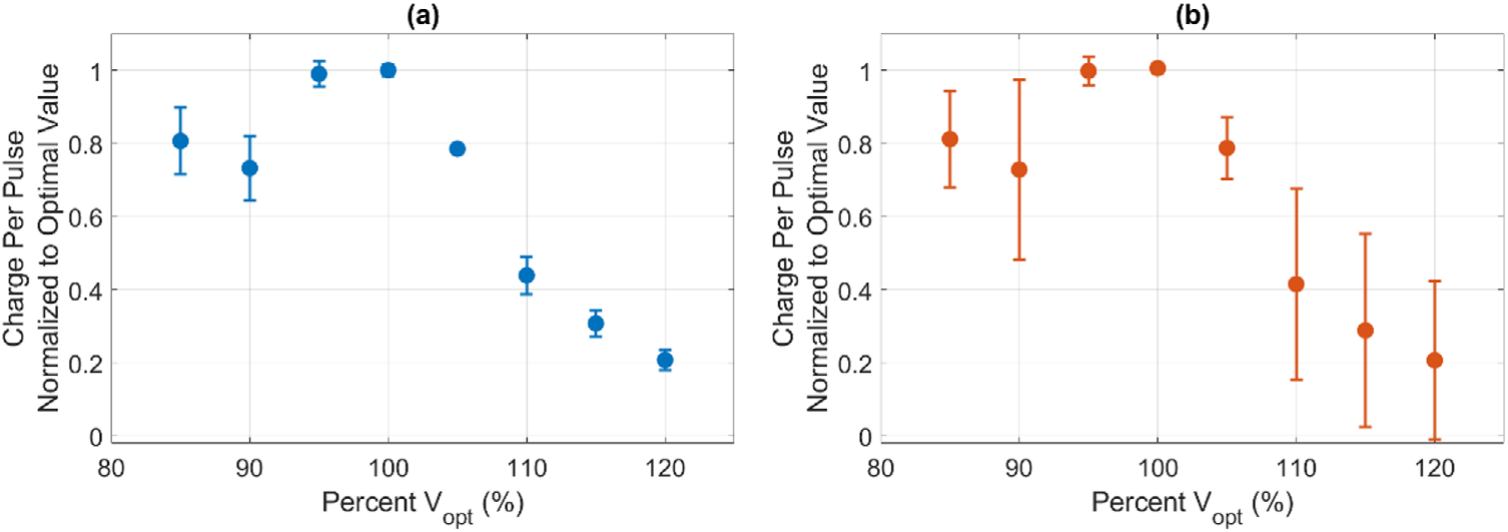
Effects on the charge per pulse of applying an external voltage (V_EXT_) to the automatic frequency controller (AFC) that deviates from the optimal voltage (V_opt_). (a) Mean charge per pulse of a 10‐pulse delivery, averaged across 5 separate deliveries (*N* = 5) and normalized to V_opt_ = 100%. (b) The corresponding results for the first pulse in each delivery in (a). Error bars represent the standard deviation of charge per pulse within each separate delivery. Both the magnitude and variance of the mean were reduced for small deviations in applied external voltage (V_EXT_) from V_opt_.

**FIGURE 6:**
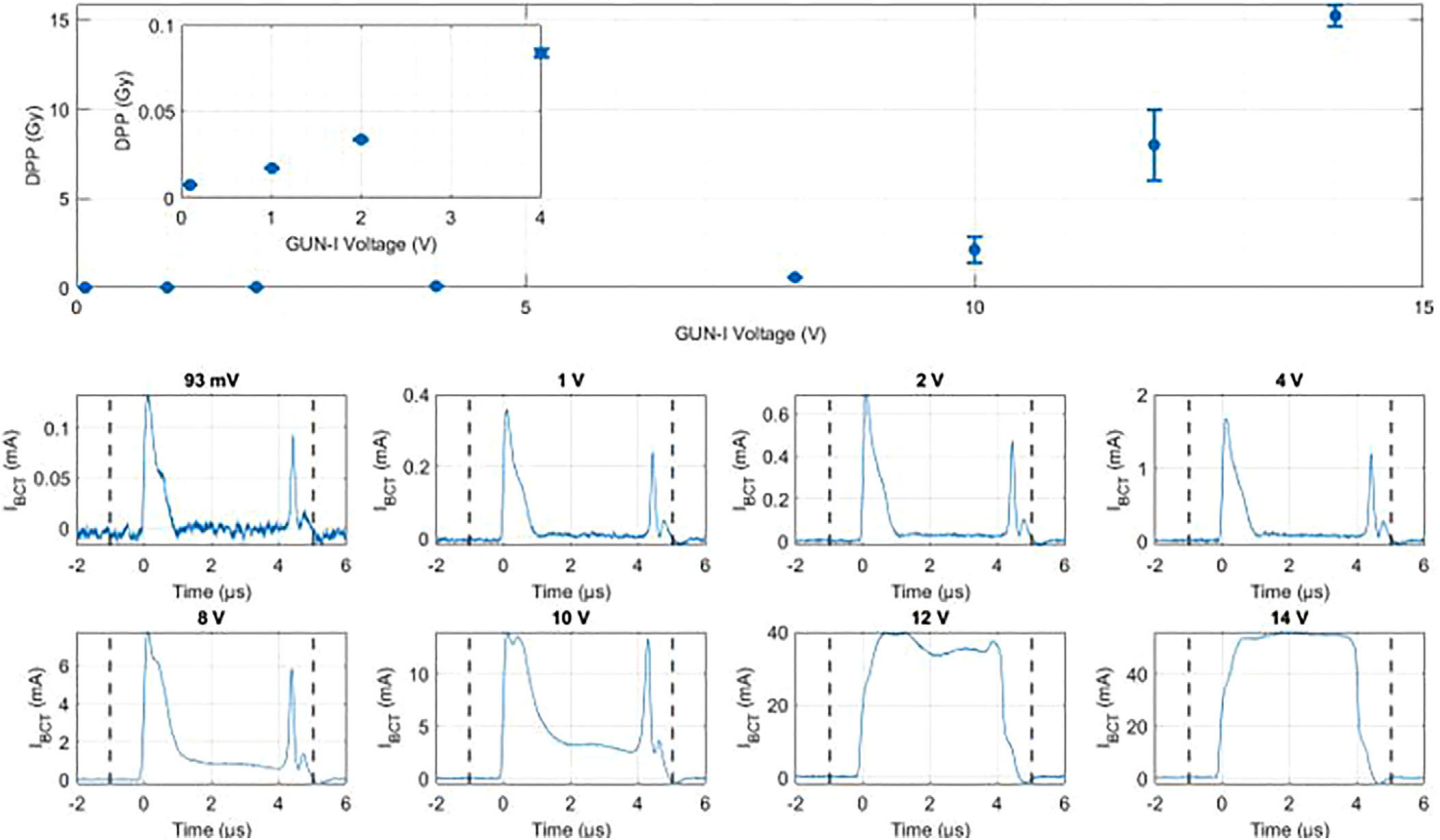
Effect of changing the gun current (GUN‐I) on the dose‐per‐pulse (DPP) and pulse shape. The inset (top) shows the relative effect for the low‐sensitivity region when GUN‐I voltages are < 4 V. Pulse shapes are shown in recorded beam current transformer current (I_BCT_) for each sampled GUN‐I value (bottom), demonstrating the change in intra‐pulse stability. Dashed lines at ‐1 µs and 5 µs representing the radiofrequency acceleration envelope^23^ demonstrate that the degradation in DPP comes from a reduced pulse height, with very minor variation in pulse width.

Published LINAC conversion procedures often report removing the X‐ray target in order to take advantage of the increased electron current in one of the photon modes.^14^, ^15^ By contrast, our method provides exceptionally high DPP while simplifying conversion process. Nevertheless, using a photon mode would present an opportunity to directly use the LINAC up to a maximum PRF of 360 Hz and thus would further increase the MDR and DPP at large SSD. The low beam‐off latency of our system (50–60 µs) suggests that the EBCS could still be used in this configuration if the re‐arming time is shorter than the pulse period at the selected PRF. Further, users may have the opportunity for some simple processing, such as signal integration or PW analysis, in the time between pulses, although this might be better enabled through processing in parallel by using a parallel system, or with a similar measurement tool and further signal splitting.

It is worth noting the limitations of this comparison. This system was developed specifically for conventional electron accelerators (6–20 MeV) capable of FLASH deliveries with pulse width and structure comparable to those used in routine clinical practice. Other electron sources, particularly those used for VHEE applications (> 100 MeV), possess unique characteristics related to pulse structure (short picosecond bunches) and transport physics. In those regimes, beam diagnostics may require gigahertz‐bandwidth systems and must account for other effects negligible at clinical energies. While the EBCS adopts non‐destructive, high‐bandwidth monitoring common to HEP, its implementation is specifically tuned to the pulse widths and sampling requirements relevant for clinically traditional accelerator designs. An additional limitation that arose with our current set‐up is the need to have long signal cables to join instruments that are inside the room with control components that are outside the room. In several instances, such as the ACCT pulse that can be seen in Figure 3, having the BCT placed a large distance from the computer oscilloscope yielded a signal impedance mismatch, which then produced oscillations in the signal that was being recorded in the brief period after the pulse.

## 5 CONCLUSIONS

We developed and validated an EBCS capable of delivering stable dose per pulse on a converted clinical linear accelerator in both conventional and ultrahigh DPP configurations, supporting both single‐ and multi‐pulse irradiations on a common platform. By implementing pre‐delivery radiofrequency optimization and controlled beam inhibition, the system enables stable beam conditions at the onset of irradiation and mitigates pulse‐to‐pulse variability. System reliability was demonstrated, with output reproducibility better than 0.5% for deliveries up to 20 pulses. The EBCS supported reproducible delivery across conventional and ultrahigh DPP regimes, highlighting its utility as a unified control framework spanning conventional and FLASH‐compatible electron beams. More broadly, this approach addresses a key limitation of clinical beam control architectures in the FLASH regime, where pulse‐resolved beam behavior directly influences delivered dose, and provides a practical pathway for improving robustness and reproducibility in electron FLASH systems based on conventional linear accelerator designs operating at clinically relevant energies (6–20 MeV).

## CONFLICT OF INTEREST STATEMENT

The authors have no relevant conflicts of interest to disclose.
